# A New Waveform to Solve the Ghosting of BWR EPD

**DOI:** 10.3390/mi16040420

**Published:** 2025-03-31

**Authors:** Yong Yang, Yunyan Xie, Jiahu Yuan, Yuehua Cui, Qiangeng Cheng, Lianghui Shi

**Affiliations:** 1 Chongqing University, Chongqing 400044, China; yy13812661769@163.com; 2 Chongqing Institute of Green and Intelligent Technology, Chinese Academy of Sciences, Chongqing 400722, China; yhcui@cigit.ac.cn; 3 Laboratory of Chongqing BOE Smart Electronics System Co., Ltd., Chongqing 400700, China; chengqiangeng@boe.com.cn (Q.C.); shilianghui@boe.com.cn (L.S.)

**Keywords:** BWR EPD, low-consumption display, ghosting, driving waveform, gradual iteration

## Abstract

The BWR (Black–White–Red) electronic paper display has the advantage of low power consumption and offers a paper-like reading experience. However, refreshing images between black and red can easily produce ghosting images. In this article, a new driving waveform is proposed, in which classic black, white, and red particles are modeled as spheres, and the Com state is introduced as a renewal element because the motion of the sphere particles must ensure momentum conservation in the BWR EPD (Electrophoresis Display) during electronic field removal. Additionally, we adopted the concepts of gradual iteration and successive promotion in the new driving waveform based on the principles of electrophoretic particle displays and combined the momentum and inertia theorem with Stokes Law. A large number of experimental data confirmed that not only was the ghosting value optimized appreciably to below 0.5, but the actual ghosting performance has been significantly improved, especially for black and red images.

## 1. Introduction

Due to the promotion of the IOT ecology and the advocacy of green environmental protection, EPD, a new type of low-carbon and environmentally friendly display device, has acquired a certain share of the display market in recent years [1,2,3,4]. Compared with traditional display devices, such as LCD, mini-LED, and Micro-LED displays, EPD offers a less colorful screen [5]. However, this technology has several advantages, including a wide viewing angle, light weight, low power consumption, re-writable data, and eye protection. Consequently, EPD technology is being rapidly applied in smart business and office devices, travel devices, smart medical treatment devices, military devices, and other intelligent products, such as electronic price tags, electronic table cards, electronic door cards, electronic bus station cards, electronic bedside cards, and electronic transfusion cards [6,7,8,9].

Although the display advantages of this technology are obvious, the key to achieving a perfect EPD display is controlling the electrophoretic display particles through the driving waveform. An electronic paper display primarily produces images as charged particles of different colors driven by different voltages. Due to the positive charge of black and red particles in the BWR EPD system, when a driving electric field applies the same directional force on them, the movement direction of black and red particles is the same, which leads to black and red mixing when the EPD is intended to display a black or red image, resulting in color ghosting. When particles in EPD are too active to maintain absolute stability under static conditions, blurring can easily occur [10,11]. Moreover, when the driving ability of particles at the edges and corners of the display image is insufficient, mura commonly appear at the edges and corners of the EPD [11,12,13]. During the shaking process, uneven shaking and the formation of particle clusters commonly produce ghosting [10,11,12,13]. This series of issues is closely related to the design of the EPD driving waveform. Therefore, designing an EPD driving waveform is particularly important to improve display and image performance.

To optimize the EPD’s imaging effects, a series of studies have been conducted. Prior researchers reduced the EPD ghosting and improved the display effects by synthesizing new materials, improving EPD particle preparation devices, and optimizing the particle-driving waveform [14,15,16]. After extensive experiments by researchers, optimizing the EPD’s driving waveform was found to significantly improve its display effects. Kao suggested using a hysteresis characteristic curve measurement method based on time delay responses to improve the display effects of EPD [17]. Johnson proposed a driving waveform with a reference waveform; in this work, the EPD driving display waveform was adjusted according to the reference. In this way, the EPD was able to display more grayscale, greatly improving display performance [18]. Lu offered a new way to reduce waveform lookup tables to accelerate the display time and ensure grayscale performance [19]. Shitao Shen optimized the driving waveform, with the fringe phenomenon quantified as a gray value that can be diminished by 25.5 while maintaining a response time of 200 ms [11]. A driving waveform based on damping oscillation was also proposed to optimize red saturation in a three-color EPD [20].

Many previous studies explored the ghosting of EPD in black and white or grayscale displays. However, few solutions have been proposed to address the issue of BWR EPD display ghosting [10,11,12,13,14,15,16,17,18,19]. In this study, a new driving waveform is proposed to resolve the ghosting problem of BWR EPD, especially black and red image ghosting. When modeling the motion modes of BWR EPD in this study, the black, white, and red particles are not regarded as “points” as in previous model designs but instead as “spheres”. By analyzing the motion state of black, white, and red particles through Stokes’ Law, combining the momentum conservation theorem, the physical characteristics of electrophoretic particles, and analysis of the force movement of particles. The Com state is also introduced into the design of the driving waveform for red particles to ensure that the charges reach equilibrium after each stage of movement and that the motion of the red particles in each stage is consistent with the preset to guarantee saturation of the red image and solve the problem of black and red ghosting in BWR EPD. The traditional waveform driving method alternately uses High (+15 V) and Low (−15 V) voltage driving in the shaking stage. In this study, we introduce the Com state into the driving waveform and divide the shaking stage into slow shaking and fast shaking to fully separate colorful particles. In addition, the fast shaking stage driving mode uses the Low (−15 V)—High (+15 V)—Com (0 V)—High (+15 V)—Low (−15 V)—Com (0 V) method to recursively push particles. In the imaging stage, the Com state is also designed using the concept of successive recursion to present a red image, and the red image is repeatedly pushed to ensure saturation of the red display image. At the same time, the refined frame rate and frequency are applied to the entire driving waveform design process to optimize ghosting and improve the imaging effects of the BWR EPD.

## 2. Principles Analysis of BWR EPD

### 2.1. The EPD Device

**Figure 1:**
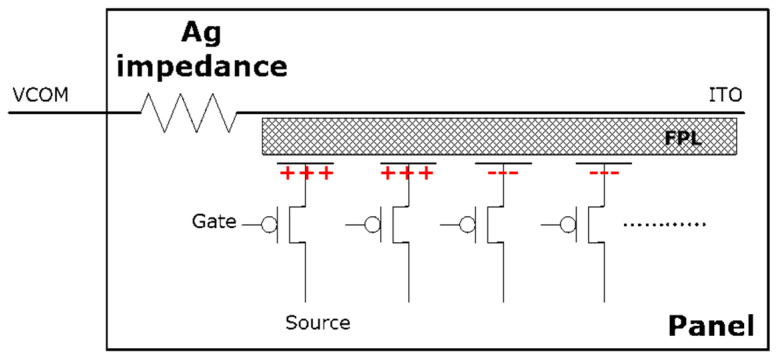
The internal circuit of the EPD.

Compared with traditional display devices, EPD displays can achieve a power-off state because the electrophoretic particles of the EPD display material, FPL (Front Plane Laminate), have bi-stable display characteristics. At present, mainstream BWR EPD screens mainly inject black, white, and red electrophoretic ink particles into a “micro cup” structure, with a large number of “micro cups” densely arranged to form electronic ink displays. The reference voltage Vcom of the FPL ITO layer and the Drain voltage of the TFT (Thin Film Transformer) ITO layer form an electric field on both sides of the “micro cup”, thereby charging particles to move up and down. After reflection, the EPD then shows the images [21,22,23]. And its internal circuit shown as Figure 1.

### 2.2. BWR EPD-Driven Particle Motion Analysis

**Figure 2:**
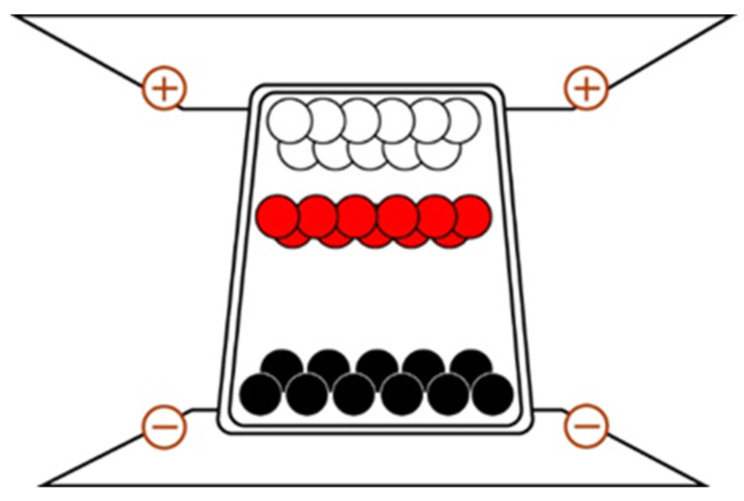
BWR particle in the “micro cup”.

Figure 2 schematically presents the motion model after modeling the black, white, and red sphere particles in the “micro cup” during the driving waveform process. Here, ITO is set as the positive and negative electrodes of EPD. The changes to the driving voltage are set based on the source of TFT, with a driving time of about 50 ms. Each pixel of the EPD image is approximately 200 um, while the gap of each pixel is about 10 um. To achieve a bi-stable EPD, the particles in the “micro cup” must maintain force balance under static conditions, otherwise the particles will float or sink. From our analysis of particle forces, particles are affected by their own gravity, the buoyancy of non-polar solvents, electrostatic force, and viscosity. When particles are in a stationary state, if they must maintain equilibrium under force, they must meet the following requirements [24]:

(1) 43πR3ρ1−ρm=0

where *R* is the particle radius, *ρ*_1_ is the density of non-polar solvents, and *ρ_m_* is the density of the particle itself.

According to Newton’s Second Law, when there are no other external forces, except for the external electric field force, particle gravity, and the buoyancy of non-polar solvents, the force acting on particles in a “micro cup” can be expressed as shown in Figure 2 [25]. If the voltage between electric fields is *U*, the charge amount is q, and the distance between electric fields is *d*. Thus, the particle’s speed and time of motion without other external forces can be expressed as follows:

(2) Uqd−6πRηv=mdvdt

where *m* is the mass of the particle, and *v* is the velocity of particle motion. Additionally, *R* is the particle radius, and *η* is the viscosity between particles and the solution. By utilizing the characteristics of first-order homogeneous linear equations, we obtain

(3) $$v=\frac{Uq}{6\pi R\mathrm{d}\eta }+C\cdot e^{-\frac{6\pi R\eta }{m}t}$$

where C is a random constant.

In practical situations, when time t is 0, the velocity *v* must be 0. Therefore, in practical applications, the above equation *C* should be a negative number. Therefore, the above equation can be expressed as

(4) $$v=\frac{Uq\left(1-e^{-\frac{6\pi R\eta t}{m}}\right)}{6\pi d\eta R}.$$

Based on an analysis of Newton’s Second Law and Stokes Law, the motion speed of particles is related to particle weight, particle radius, particle charges, driving time, and particle driving voltage. Additionally, the speed of particle motion affects the position of particles after they are stationary, thereby affecting the display effects of EPD images. Therefore, the display effects of EPD images are determined by particle mass, radius, charge, driving time, and driving voltage.

## 3. An Improved Method for a BWR EPD Driving Waveform 

Three colors of electrophoretic particles exist in the electronic ink displays of FPL: black, white, and red. Black particles are manufactured by modifying carbon black and have a positive charge. White particles are manufactured by modifying titanium dioxide and have a negative charge. Red particles are manufactured by compounds such as iron oxide and also have a positive charge. Since black particles and red particles have the same type of charge, if the two particles are driven by the same voltage and have similar nuclear/cytoplasmic ratios, the motion speed of the two particles will also be similar. However, it remains difficult to separate the two particles, leading to ghosting in imaging displays. To distinguish between two colored particles, the weight of red particles will be greater than that of black particles when preparing the particles. Equation (4) shows that under the same conditions, black particles have a light weight, affording them fast motion. At the same time, when designing the driving waveform, the driving voltage of black particles is greater than that of red particles, further accelerating the movement speed of black particles. Through the differences in particle characteristics and the voltage design of the driving waveform, the motion speed of red and black electrophoretic particles can be differentiated and used to display black and red images.

The TFT and source IC (Integrated Circuit) support between ±2.4 V and ±15 V as driving voltage, while the black and white particle driving voltage has the opposite polarity. Therefore, the corresponding voltages of black and white particles range from 2.4 V to 15 V and from −2.4 V to −15 V, respectively. According to the physical characteristics of red particles, when the driving voltage is greater than 8.8 V, red particles are difficult to push. Therefore, the driving voltage of red particles ranges from 2.4 V to 8.8 V. Due to the same polarity between black and red particles, if the driving voltage of the two is similar and below 8.8 V, black and red particles can be easily confused. The weight, radius, and charge of the particles cannot be changed in an electric field device, and the spacing between electric fields is fixed. According to Equation (4), when the driving waveform is designed, the larger the driving voltage is, the faster the particle moves. Therefore, we applied driving voltages of +15 V and −15 V for black and white particles, respectively. A large number of experiments verified that if the red driving voltage is lower than 4 V, it faces difficulties in driving red particles. When the driving voltage is higher than 7 V, black and red particles can easily move together, leading to confusion in the black and red images. Here, the red particle driving voltage is between 4 V and 7 V. In addition, red particles have a larger nuclear/cytoplasmic ratio compared to that of black and white particles and are more easily driven.

**Figure 3:**
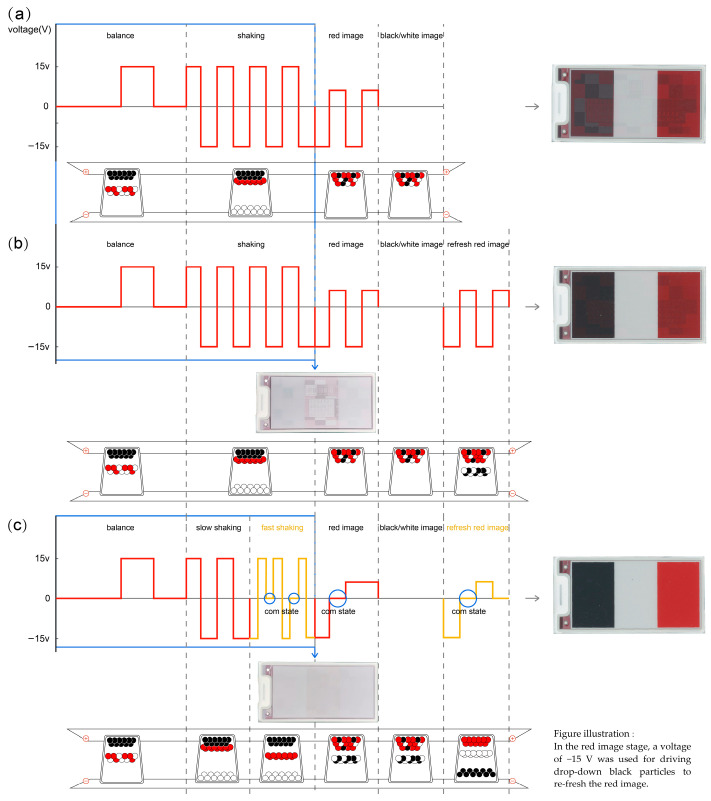
(**a**) Traditional driving waveform. (**b**) Waveform with the addition of refreshed red particles. (**c**) New waveform.

Based on the above analysis, each particle color must provide a certain voltage under a certain electric field to drive and display the image. The process of red-particle-driven waveform image formation involves three main stages: balance, shaking, and imaging. Figure 3a presents the various driving waveforms and their display images. Figure 3a illustrates that the ghosting of black and red particles is serious as the shaking and imaging stages are not sufficient.

To solve the ghosting problem of BWR EPD, we added an imaging stage for red particles and designed a new driving waveform, as shown in Figure 3b. After adding the imaging stage of red particles, the ghosting of black and red particles was improved, as shown in Figure 3b, but the image performance was not yet satisfactory.

The shaking stage mainly involved dispersing particles. We found that employing a slow shaking speed could enable us to distinguish between heterogeneous charge particles located at the two poles of the substrate due to having enough time for particle movement and the same type of charged particles separated via a fast shaking speed due to different charge quantities in different colorful particles with different moving speeds. Therefore, in the newly designed driving waveform, we divided the shaking process into slow shaking and fast shaking. Slow shaking mainly involved shaking and dispersing the white particles between black and red particles in a “micro cup”. According to the law of conservation of momentum and analysis of the driving waveform and its display performance, fast shaking used the Low (−15 V)—High (+15 V)—Com (0 V)—High (+15 V)—Low (−15 V)—Com (0 V) method, mainly by shaking and dispersing the black and red particles in the “micro cup”. The Com state confirmed the particles’ positions during fast shaking. This shaking method could also help separate colorful EPD particles under the same type of charge. The imaging stage also adopted the idea of successive recursion, adding a Com (0 V) state to fully disperse black, white, and red electrophoretic particles. At the same time, the imaging stage added a stage of refreshing the red image again to eliminate black ghosting and ensure saturation of the red image. The new driving waveform is shown in Figure 3c. Here, we can see that the performance of the new driving waveform is perfect.

## 4. Results and Discussion

### 4.1. Software Setting Parameters and Conditions

In the driving waveform design, the driving voltage, driving time, driving frequency, and waveform layout of black–white–red particles were optimized. The experimental parameters were set as follows. Under room temperature (298.15 ± 3 K) conditions, the slow shaking frequency was set to 50 Hz, and the fast shaking frequency was set to 100 Hz. The driving voltages for black and white particles were +15 V and −15 V, respectively. Additionally, the driving voltage for red particles was set to +6.4 V, which was able to fulfill the driving requirements of BWR particles.

### 4.2. Driving Waveform Design

**Figure 4:**
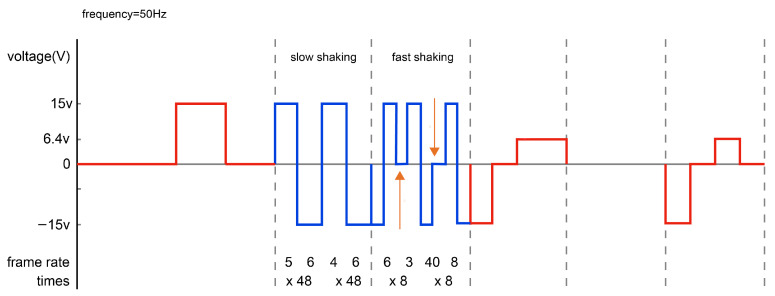
BWR particles in the “micro cup”.

**Figure 5:**
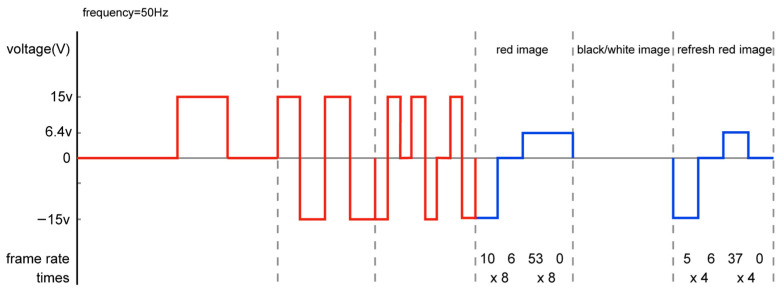
The new waveform of red particles in the imaging stage.

Next, we conducted a detailed experimental analysis on introducing a Com state into the driving waveform of red particles during the fast shaking and imaging stages. The actual driving waveform designs for fast shaking were determined after comparing multiple sets of experimental data (with different frames and different rates). These designs are shown in blue in Figure 4, while the imaging stages are shown in blue in Figure 5. 

The shaking stage involved slow shaking first, followed by fast shaking. Additionally, fast shaking adopted the Low (−15 V)—High (+15 V)—Com (0 V)—High (+15 V)—Low (−15 V)—Com (0 V)) mode, and the number of shaking times gradually recursed. Specific implementation involved (1) slow shaking with five and six frames for BWR particles, followed by (2) slow shaking with four and six frames. We then (3) repeated the operation 48 times. To ensure that the black and red particles were shaken evenly and dispersed, a Com dwell action was added in the middle of fast shaking, as shown with an orange arrow in Figure 4. This process was carried out by (1) using six and three frames for fast shaking, followed by (2) fast-shaking with forty and eight frames. Then, we (3) repeated the operation eight times, as shown in Figure 4.

Imaging stage: To ensure saturation of the red display, the imaging stage first involved pushing the red image and then imaging the black and white image, followed by imaging the red image again. Similarly, a recursive design was used in the red imaging stage. The first red image used the Low (−15 V)—Com (0 V)—High (+6.4 V)—High (+6.4 V) mode, and the second red image used the Low (−15 V)—Com (0 V)—High (+6.4 V) mode. The specific implementation process for the initial imaging of the red image was as follows: (1) After pushing ten frames of black particles, (2) we paused six frames, (3) pushed 53 frames of red particles again, and (4) repeated the operation eight times. Imaging the red image again involved (1) pushing five frames of black particles, followed by (2) pausing for six frames and (3) pushing 37 frames of red particles. We then (4) repeated the operation four times.

### 4.3. Equipment and Methods in the Experiment

**Figure 6:**
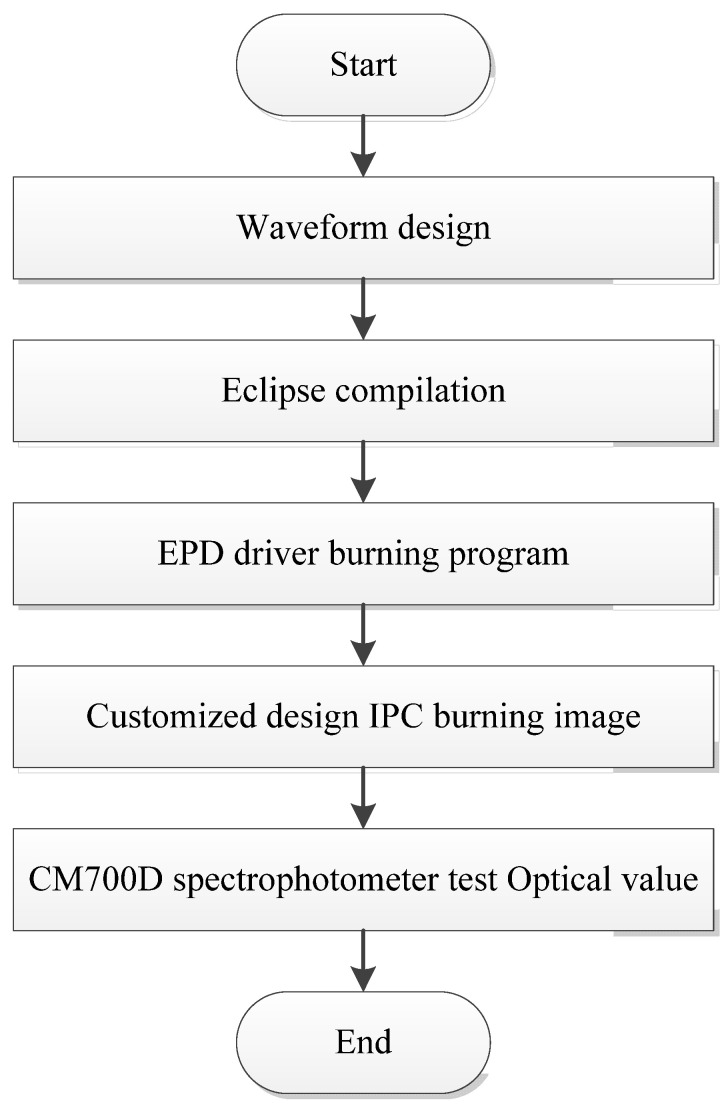
The new waveform of red particles in the imaging stage.

To verify the actual display effects of the designed driving waveform, we compiled the novel waveform in the Eclipse compilation environment and output the waveform file. An EPD driver burning board was used to burn the compiled bin file into EPD. BWR EPD refreshing and imaging were performed using customized IPC tools and an EPD refreshing board. IPC was designed for the imaging data process and transformation along with the EPD refreshing board responsible for receiving imaging data and driving EPD to the display. The hardware system and equipment required for EPD ghosting testing included a CM700D spectrophotometer (Konica Minolta, Tokyo, Japan), an EPD refreshing board, and a custom IPC. The testing process shown in Figure 6 included (1) zero calibration and whiteboard calibration on a CM700D spectrophotometer. Next, we (2) applied the sample image onto the solid color image (black, white, or red image), (3) waited for 20 s, and (4) used a CM700D contact alignment to test the center of the EPD image. Then, we (5) recorded optical test data. Finally, through a data comparison, we confirmed whether the designed driving waveform file could achieve the display effect. The criteria for determining EPD ghosting were as follows: ghosting was invisible upon visual inspection; if ghosting was visible, it had to agree with the following CIEDE 2000 color difference formula ΔE (Ghosting Value) < 1. 

### 4.4. Experimental Data and Comparison

**Figure 7:**
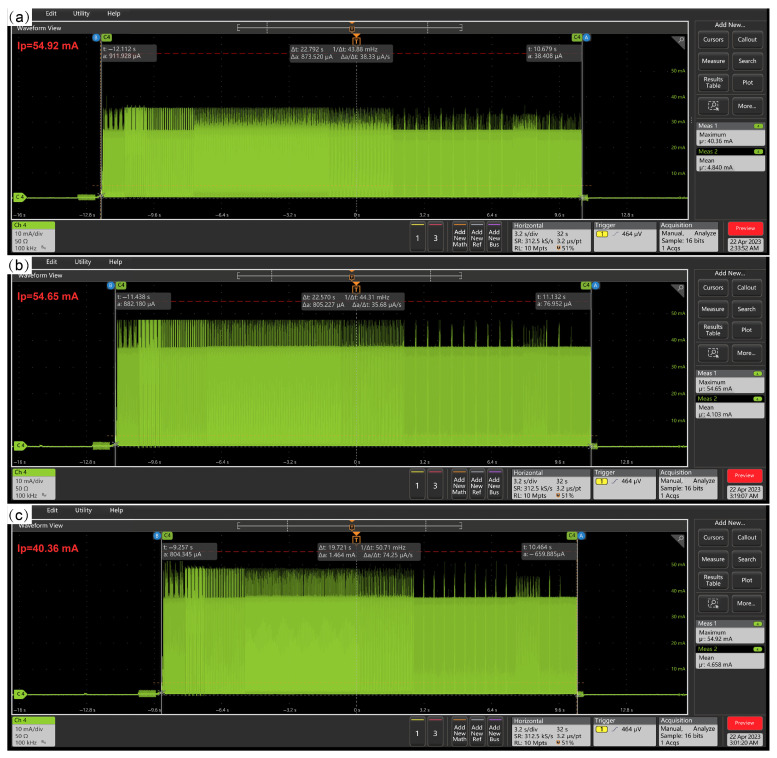
(**a**) The current waveform of traditional driving waveform. (**b**) The current waveform of refresh red particle waveform. (**c**) The current waveform of new waveform.

At room temperature (298.15 ± 3 K), we burned the above three driving waveform bins for the same 2.66″ BWR EPD and used a Tektronix MSO44 mixed signal oscilloscope (Tektronix, Beaverton, OR, USA) and Tektronix TCP A300 current probe (Tektronix, Beaverton, OR, USA) to test the corresponding current, as shown in Figure 7. The peak current of the traditional driving waveform was 14.56 mA higher than that of the new driving waveform. Therefore, adding a Com state to the driving waveform can effectively reduce the peak current during the driving process.

**Figure 8:**
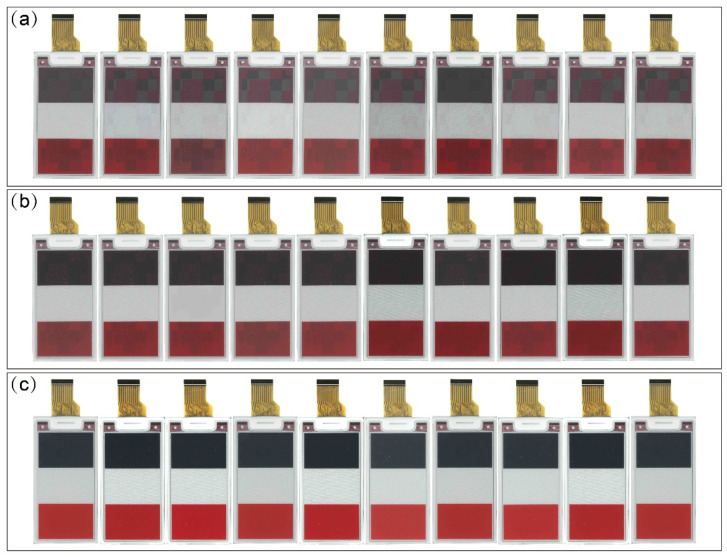
(**a**) BWR EPD ghosting in the traditional waveform. (**b**) BWR EPD ghosting in the waveform with refreshed red particles. (**c**) BWR EPD ghosting in the new waveform.

**Figure 9:**
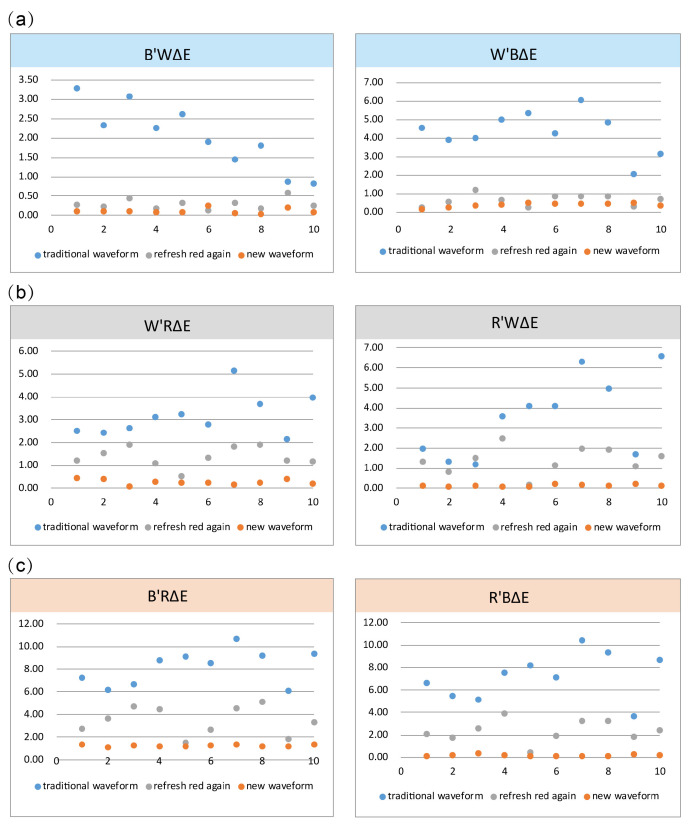
(**a**) The ghosting data for the black to white image and the white to black image. (**b**) The ghosting data for the white to red image and the red to white image. (**c**) The ghosting data for the black to red image and the red to black image. Blue dots represent the values of ghosting in traditional waveforms, grey dots represent the value of ghosting in the waveform, including refreshed red particles, and orange dots represent the value of ghosting in the new waveform.

We randomly selected 10 pieces of 2.66 inch BWR EPD for experimental verification. We then burned the newly designed driving waveform program and traditional driving waveform into the same samples and compared the performance results. The ghosting of the newly designed driving waveform was found to be significantly improved in actual verification testing. Additionally, when converting black and red images, the ghosting values (B’RΔE and R’BΔE) were reduced from more than 5 to less than 0.3. When refreshing images, the ghosting was significantly improved in BWR EPD, as shown in Figure 8. We calculated the test data using color difference formula CIEDE 2000, and all ghosting data are shown in Figure 9. Here, when converting black and white images, the ghosting value (B’WΔE and W’BΔE) was reduced to less than 0.5. When converting white and red images, the ghosting value (W’RΔE and R’WΔE) was reduced to less than 0.5.

## 5. Conclusions

Research and experiments show that our newly developed driving waveform based on the law of conservation of momentum offers a novel method for red sphere particles by introducing Com Status and adopting the idea of gradual iteration and successive promotion in the fast shaking and red image imaging stages. Additionally, for the driving waveform, we separated the shaking stage into slow shaking and fast shaking using the Low (−15 V)—High (+15 V)—Com (0 V)—High (+15 V)—Low (−15 V)—Com (0 V) method. This method enabled us to fully separate colorful particles and could guide the separation of other colorful particles. Moreover, we added a red image waveform design during the red imaging phase, as well as a Com mode. In the overall driving waveform design, each stage promoted the frame refinement design of BWR electrophoresis particles. Using the designed driving waveform, not only was the ghosting value optimized to below 0.5, but the actual ghosting performance was also significantly improved. Importantly, the pulse current was also much lower in the driving waveform process.

## Data Availability

The original contributions presented in the study are included in the article, further inquiries can be directed to the corresponding authors.
